# Estimating and Analyzing Long-Term Multi-GNSS Inter-System Bias Based on Uncombined PPP

**DOI:** 10.3390/s20051499

**Published:** 2020-03-09

**Authors:** Fan Zhang, Changjian Liu, Guorui Xiao, Xi Zhang, Xu Feng

**Affiliations:** Information Engineering University, Zhengzhou 450001, China; chxylcj@163.com (C.L.); 13903781995@139.com (X.Z.); Geodesy_fx@163.com (X.F.)

**Keywords:** uncombined model, inter-system bias, multi-GNSS, receiver type, precise point positioning

## Abstract

With the development of precise positioning with multi-GNSS, the inter-system bias (ISB) has become an issue that cannot be ignored. ISB is introduced from the differences among satellite reference clocks and different receiver hardware delay biases. To analyze the characteristics of multi-GNSS ISB, the precise point positioning (PPP) with full-rank uncombined model was derived for GLONASS, BDS, GALILEO, while the GPS receiver clock was selected as the reference. In addition, a recommended ISB parameter processing model was adopted. Data of 28-days from the Multi-GNSS Experiment (MGEX) station was used to estimate and analyze the ISB parameters. Based on a statistical analysis of the acquired data, results demonstrate that: (a) The rms of multi-GNSS PPP positional bias can reach 4.6 mm, 3.4 mm and 8.5 mm for E, N and U directions, respectively, which guarantees the reliability and accuracy of the ISB parameter solution. (b) The intra-day ISB time series of the three groups is relatively stable with standard deviations less than 0.6 ns. The ISB parameters between the GALILEO and GPS system are the most stable and the standard deviation was the smallest, at about 0.37 ns, which may be related to the good signal quality of the GALILEO system. (c) The mean of the single-day solution of the ISB parameter is not stable and the amplitude of the jump can be up to 60 ns. However, each station shows a similar variation for the same ISB parameter on the same day. The situation is independent of the type of receiver and antenna; however, it may be affected by the satellite reference clock of different systems. (d) There is a clear relationship between the ISB parameters and receiver types.

## 1. Introduction

With the implementation and improvement of multi-GNSS constellations, precise point positioning (PPP) is rapidly developing from traditional GPS-only dual frequency systems to multi-system-multi-frequency systems. PPP is widely used in many areas, e.g., seismic rescue and ocean exploration [1,2,3,4,5]. As of 4 June 2019, there are 115 globally distributed satellites form the four different systems that can provide user location services. GPS and GLONASS, as completed constellation systems, can provide comprehensive GPS timing navigation (PNT) services [6]. Built in 1999, the GALILEO system represents a new generation of civilian global satellite navigation systems. The full GALILEO system constellation will consist of 30 satellites and will distribute over three orbital planes. Currently, 26 medium Earth orbit(MEO) satellites are in orbit to provide positioning and navigation services. GALILEO satellites transmit signals at five different frequencies, i.e., E1 (1575.42 MHz), E5a (1176.45 MHz), E5b (1207.14 MHz), E5ab (1191.795 MHz) and E6 (1278.75 MHz). The BeiDou Navigation Satellite System (BDS) is the first navigation system [7] in which all satellites transmit tri-frequency signals, i.e., B1 (1561.098 MHz), B2 (1207.14 MHz) and B3 (1268.52 MHz). Currently, there are 33 BDS satellites at the working phase and another six (1GEO + 3IGSO + 2MEO) are under the in-orbit test phase [8].

When jointly processing multi-GNSS data, it is necessary to properly deal with the different receiver clocks. Existing methods could be generally divided into two types. The first type selects a reference system (mostly GPS), and only a single receiver clock difference parameter is built, and the ISB parameter is generated at the same time [9,10]; The second approach sets separate receiver clock parameters for each system [11]. The advantage of the second approach is that ISB is not considered, and parameters to be estimated are not increased or decreased. With regard to the ISB parameter, when we select GPS as the reference system, the time reference is aligned to the GPS time in the joint processing function model; however, for multiple systems, the satellite reference clocks selected in the calculation process still differ, which also results in differences of the reference clock [12]. In addition, because of the satellite type, standard, frequency and other system specifications, the signals will be processed through different channels when the signals are received. This means that the signal hardware delays of different satellite systems will be inconsistent, relative to the receiver [13,14]. In summary, ISB parameters are related to satellite reference clock differences and hardware delay biases, due to different receiver configurations.

Research has been conducted to investigate ISB by single point positioning (SPP) technology. In the SPP function model, ISB parameters also include the two elements mentioned above [13]. Torre et al. studied the ISB parameters between the GLONASS, GALILEO, BDS and GPS systems using SPP, and analyzed its influence on the positioning results [15]. However, the accuracy was poor, and only the variation of ISB on a single day were analyzed. Zeng et al analyzed the characteristics of BDS–GPS ISB using SPP and designed a statistical method based on a hypothesis test to evaluate the stability of ISB parameters. Results confirmed the correlation between ISB parameters and receiver type [13]. However, their method only includes the ISB between BDS and GPS, while that of the GALILEO and GLONASS were not considered. In addition, a long-term ISB instead of a single day analysis should be conducted. Cai and Gao used SPP technology to investigate the ISB parameters between GLONASS and GPS from five international GNSS service (IGS) tracking stations. Results indicated that, the variation of ISB parameter can be up to 40 ns over a single day [16]. Chen et al., studied the relationship between the globally distributed IGS tracking station receivers and ISB parameters [17]. The main problem of the SPP approach is that the accuracy is relatively low. Nevertheless, after correcting the ISB parameter, the SPP positioning accuracy of multiple systems can still be improved [18].

With the development and application of PPP technology, more researchers have started to use PPP technology to investigate the ISB parameter [19,20,21] or have adopted multi-system PPP with ISB corrected for practical application [22,23]. Wang et al., proposed a four-system combined PPP algorithm with ISB parameters based on GPS, GLOASS, GALILLEO and BDS. Based on the ionosphere-free combination model, this algorithm obtains the ISB parameters of between GLONASS, BDS, GALILEO and GPS through a static processing of seven Multi-GNSS Experiment (MGEX) tracking stations data. The results demonstrate that ISB shows a moderate slight intra-day variation that is related to the receiver type. The multi-day ISB time series also includes irregular jumps, which can be up to 20 ns [12]. However, this algorithm is based on the ionospheric-free combination model, and the process noise is amplified by approximately three times, which may affect ISB parameter estimation. In addition, this method is not applicable to ISB estimation and analysis on a day when the ionosphere changes significantly. Zhou et al., studied multi-GNSS PPP considering ISB parameters based on an uncombined model and discussed different processing strategies for ISB parameters, however, the characteristics of ISB parameters were not investigated or analyzed [19]. Guo et al., applied multi-system PPP considering ISB in precision agriculture. Compared to the single GPS system, the dynamic and static positioning accuracy are decreased from 19 mm and 126 mm, to 6 mm and 35 mm, respectively, and ISB parameters were estimated as a constant within a day [24]. Up to now, a detailed long-term analysis and comparison of ISB parameters of the four systems based on the uncombined model has not been conducted.

To analyze the characteristics of multi-system ISB parameters, the four-system uncombined PPP function model was first deduced, followed by the specific definitions of ISB parameters. It is worth noting that we specifically consider the processing of the pseudo range frequency deviation of the GLONASS system. After formula derivation and separation, we set an inter-frequency-biase (IFB) parameter for each epoch, which is conducive to eliminating the influence of pseudo range IFB parameter on positioning. Compared with the method of setting the IFB parameter one by one for the GLONASS satellite, the model produced in the manuscript can greatly reduce the number of parameters to be estimated for each epoch, greatly enhance the structural strength of the model, and improve the efficiency of model estimation. We then introduce the experimental scheme and data processing method. First, we analyze the advantages of multi-system PPP in the number of available satellites, dilution of precision (DOP) value and positioning accuracy, so as to provide a better reliability guarantee for the analysis of ISB parameter characteristics in subsequent experiments. Next, the intra-day and inter-day variation characteristics of three groups of ISB parameters and their relationship with the receiver type of the station are presented in detail. Finally, conclusions and future prospects are summarized.

## 2. Multi-GNSS Uncombined Model

In this chapter, we mainly carry out the deduction of the four-system PPP uncombined function model. Aimed at the problem that there are many parameters to be estimated for the four system uncombined PPP and the inter-frequency deviation of the pseudo range of the GLONASS system, we separate the pseudo range IFB parameter of GLONASS system of the single epoch through theoretical derivation, simplify the PPP function model, and improve the model strength and parameter estimation efficiency. Then, we discuss the form of ISB parameters and the parameter estimation model, which provide the basis for the following experiments.

### 2.1. General GNSS Observation Model

The raw GNSS observation equation can be expressed as follows:

(1) $$\left\{ \begin{array}{l} P_{r,i}^{j,S}=\rho_{r}^{j,S}+c\cdot \left(\delta t_{r}-\delta t^{j,S}\right)+T_{r}^{j,S}+u_{i}^{S}\cdot I_{r,1}^{j,S}+c\cdot B-c\cdot b_{i}^{j,S}+d_{rel}^{j,S}+\varepsilon_{i,P}^{j,S}\\ L_{r,i}^{j,S}=\rho_{r}^{j,S}+c\cdot \left(\delta t_{r}-\delta t^{j,S}\right)+T_{r}^{j,S}-u_{i}^{S}\cdot I_{r,1}^{j,S}+\lambda_{i}^{S}\cdot M_{r,i}^{j,S}+d_{rel}^{j,S}+\varepsilon_{i,P}^{j,S} \end{array}\right.$$

where, j,r,i,S denote the satellite pseudo random noise code (PRN)number, receiver, frequency (i=1,2) and system (G/E/C/R), respectively. If (S = R), then $B=b_{r,i}^{j,S}$, if S = (G, C, E), then $B=b_{r,i}^{S}$; $P_{r,i}^{j,S}$ and $L_{r,i}^{j,S}$ represent pseudorange and carrier phase observations in meters, respectively, $\rho_{r}^{j,S}$ indicates the geometric distance from the satellite to the receiver and $\delta t_{r}$ and $\delta t^{j,S}$ are the receiver and satellite clock differences, respectively. c represents the speed of light in a vacuum, $T_{r}^{j,S}$ indicates tropospheric delay, $u_{i}^{S}={\left(\frac{f_{1}^{S}}{f_{i}^{S}}\right)}^{2}$ represents the ionospheric coefficient factor and $I_{r,1}^{j,S}=\frac{40.3}{{\left(f_{1}^{S}\right)}^{2}}sTEC_{r}^{j,S}$ represents the ionospheric parameters of the first frequency. $M_{r,i}^{j,S}$ represents the ambiguity including hardware delay and initial phase deviation of the satellite and receiver on the frequency i of satellite j, $\lambda_{i}^{S}$ denotes the wavelength of the *i*-th frequency, $b_{r,i}^{j,S}$ and B are pseudorange hardware delays at the receiver and satellite, respectively, $d_{rel}^{j,S}$ represents the relativistic effect error and $\varepsilon_{i,P}^{j,S}$, $\varepsilon_{i,L}^{j,S}$ represent pseudorange and carrier phase observation noise including multipath error, respectively.

When using the precise ephemeris products provided by IGS, the relationship with the true satellite clock difference is presented as [25]: $\delta {\tilde{t}}^{j,S}=\delta t^{j,S}-\frac{u_{2}^{s}}{u_{2}^{s}-1}\cdot b_{1}^{j,S}+\frac{1}{u_{2}^{s}-1}b_{2}^{j,S}+dD^{S}$, where, $dD^{S}$ represents the bias of the reference clock, which is independent of the receiver and introduced by the precise clock product. Models were used to correct the tropospheric dry component, relativistic effect and tides effect. Then, the calculated corrections were subtracted from the observations to obtain the following expression (S=G,C,E) based on Equation (1):

(2) $$\begin{array}{l} \left\{ \begin{array}{l} \Delta P_{r,i}^{j,S}=-\gamma_{r,i}^{j,S}\cdot \Delta r+c\cdot \delta {\tilde{t}}_{r}^{S}+M_{w}^{S}\cdot T_{w}+u_{i}^{S}\cdot {\tilde{I}}_{r,1}^{j,S}+\varepsilon_{i,P}^{j}\\ \Delta L_{r,i}^{j,S}=-\gamma_{r,i}^{j,S}\cdot \Delta r+c\cdot \delta {\tilde{t}}_{r}^{S}+M_{w}^{S}\cdot T_{w}-u_{i}^{S}\cdot {\tilde{I}}_{r,1}^{j,S}+\lambda_{i}^{S}\cdot {\tilde{M}}_{r,i}^{j,S}+\varepsilon_{i,L}^{j} \end{array}\right. \end{array}$$

The variables in Equation (2) are expressed as follows: The equivalent ionosphere at the first frequency: ${\tilde{I}}_{r,1}^{j,S}=\frac{40.3}{{\left(f_{1}^{S}\right)}^{2}}\cdot sTEC_{r}^{j,S}-\frac{c}{u_{2}^{S}-1}\left(DCB_{12}^{j,S}+DCB_{r,12}^{j,S}\right)$; The equivalent ambiguity parameter: ${\tilde{M}}_{r,i}^{j,S}=M_{r,i}^{j,S}+\frac{c}{\lambda_{i}^{S}\left(u_{2}^{S}-1\right)}\left(DCB_{12}^{j,S}+DCB_{r,12}^{j,S}\right)-\frac{c}{\lambda_{i}^{S}}\left(\frac{u_{2}^{S}}{u_{2}^{S}-1}\cdot b_{r,1}^{j,S}-\frac{1}{u_{2}^{S}-1}b_{r,2}^{j,S}\right)-\frac{c}{\lambda_{i}^{S}}\left(\frac{u_{2}^{S}}{u_{2}^{S}-1}b_{1}^{j,S}-\frac{1}{u_{2}^{S}-1}b_{2}^{j,S}\right)$; difference phase bias (DCB) parameters of satellite and receiver: $DCB_{12}^{j,S}=b_{1}^{j,S}-b_{2}^{j,S};DCB_{r,12}^{j,S}=b_{r,1}^{j,S}-b_{r,2}^{j,S}$; vector of position deviation parameter: $\Delta r={\left(\begin{array}{ccc} \Delta x & \Delta y & \Delta z \end{array}\right)}^{T}$; Receiver clock: $\delta {\tilde{t}}_{r}^{S}=\delta t_{r}+D_{r,IF}^{S}+dD^{S}$; ionosphere-free combination of receiver hardware delay: $D_{r,IF}^{S}=\frac{u_{2}^{S}}{u_{2}^{S}-1}\cdot b_{r,1}^{S}-\frac{1}{u_{2}^{S}-1}b_{r,2}^{S}$.

Unlike the GPS system, which uses code division multiple access (CDMA), the GLONASS system uses frequency division multiple access (FDMA) technology, which introduces obvious differences between different satellite frequencies (IFB). It can be seen from Equation (2) that the receiver pseudo-range hardware delay $b_{r,i}^{j,S}$ of GLONASS system is related to satellite number, while the $b_{r,i}^{S}$ parameters of the other three GNSS systems are independent of satellite number. Generally, IFB can be divided into carrier IFB and pseudo-range IFB, in which the carrier IFB parameters are usually absorbed by the ambiguous parameters and can be ignored in the float solution of PPP. The pseudo-range IFB of GLONASS system is usually modeled as a function of frequency number, and then estimated together with other parameters. However, this may introduce a large number of IFB parameters related to satellite or satellite frequency numbers into the equation. Considering that there are already a large number of parameters to be estimated in the four-system uncombined PPP, the stability and efficiency of the normal equation will be significantly affected by this method. Studies show that the pseudo-range hardware delay of the GLONASS system is approximately linearly correlated with the frequency number $\eta^{j}$ [26,27], and the pseudo-range observations are generally given much smaller weights compared with carrier phase equations, so the pseudo-range IFB parameters can be approximately set as follows:

(3) $$IFB=b_{r,i}^{j,S}-b_{r,i}^{\beta ,S}=\eta^{j}\cdot \Pi_{r,i}^{R}$$

In Equation (3), the pseudo-range IFB parameter is obtained based on pseudo-range hardware delay of satellite β of which frequency number $\eta^{j}$ is zero. Then the model of the GLONASS system can be expressed as follows:

(4) $$\left\{ \begin{array}{l} \Delta P_{r,1}^{j,R}=-\gamma_{r,1}^{j,R}\cdot \Delta r+c\cdot \delta {\tilde{t}}_{r}^{R}+M_{w}^{R}\cdot T_{w}+u_{1}^{R}\cdot {\tilde{I}}_{r,1}^{j,R}+\varepsilon_{1,P}^{j}\\ \Delta P_{r,2}^{j,R}=-\gamma_{r,2}^{j,R}\cdot \Delta r+c\cdot \delta {\tilde{t}}_{r}^{R}+M_{w}^{R}\cdot T_{w}+u_{2}^{R}\cdot {\tilde{I}}_{r,1}^{j,R}+c\cdot \eta^{s}\cdot \Pi_{r}^{R}+\varepsilon_{2,P}^{j}\\ \Delta \Phi_{r,1}^{j,R}=-\gamma_{r,1}^{j,R}\cdot \Delta r+c\cdot \delta {\tilde{t}}_{r}^{R}+M_{w}^{R}\cdot T_{w}-u_{1}^{R}\cdot {\tilde{I}}_{r,1}^{j,R}+\lambda_{1}^{R}\cdot {\tilde{M}}_{r,1}^{j,R}+\varepsilon_{1,\Phi }^{j}\\ \Delta \Phi_{r,2}^{j,R}=-\gamma_{r,2}^{j,R}\cdot \Delta r+c\cdot \delta {\tilde{t}}_{r}^{R}+M_{w}^{R}\cdot T_{w}-u_{2}^{R}\cdot {\tilde{I}}_{r,1}^{j,R}+\lambda_{2}^{R}\cdot {\tilde{M}}_{r,2}^{j,R}+\varepsilon_{i,\Phi }^{j} \end{array}\right.$$

${\tilde{M}}_{r,1}^{j,R}$ represent the equivalent ambiguity of each frequency, the variables in Equation (4) are expressed as follows:

(5) $$\left\{ \begin{array}{l} {\tilde{I}}_{r,1}^{j,R}=\frac{40.3}{{\left(f_{1}^{R}\right)}^{2}}\cdot sTEC_{r}^{j,R}-\frac{c}{u_{2}^{S}-1}\left(DCB_{12}^{j,R}+DCB_{r,12}^{R}\right)+c\cdot \eta^{j}\cdot \Pi_{r,1}^{R}\\ {\tilde{M}}_{r,i}^{j,R}=M_{r,i}^{j,R}+\frac{c}{\lambda_{i}^{R}\left(u_{2}^{R}-1\right)}\left(DCB_{12}^{j,R}+DCB_{r,12}^{j,R}\right)-\frac{c}{\lambda_{i}^{R}}\left(\frac{u_{2}^{R}}{u_{2}^{R}-1}\cdot b_{r,1}^{j,R}-\frac{1}{u_{2}^{R}-1}b_{r,2}^{j,R}\right)\\ -\frac{c}{\lambda_{i}^{R}}\left(\frac{u_{2}^{R}}{u_{2}^{R}-1}b_{1}^{j,R}-\frac{1}{u_{2}^{R}-1}b_{2}^{j,R}\right)+\frac{c\cdot u_{i}^{R}\cdot \eta^{j}\cdot \Pi_{r,1}^{R}}{\lambda_{i}^{R}}\\ DCB_{12}^{j,R}=b_{1}^{j,R}-b_{2}^{j,R};DCB_{r,12}^{S}=b_{r,1}^{\beta ,R}-b_{r,2}^{\beta ,R}\\ \Delta r={\left(\begin{array}{ccc} \Delta x & \Delta y & \Delta z \end{array}\right)}^{T}\\ \delta {\tilde{t}}_{r}^{R}=\delta t_{r}+D_{r,IF}^{R}+dD^{R}\\ D_{r,IF}^{R}=\frac{u_{2}^{R}}{u_{2}^{R}-1}\cdot b_{r,1}^{\beta ,R}-\frac{1}{u_{2}^{R}-1}b_{r,2}^{\beta ,R}\\ \Pi_{r}^{R}=\Pi_{r,2}^{R}-u_{2}^{R}\cdot \eta^{j}\cdot \Pi_{r,1}^{R} \end{array}\right.$$

### 2.2. Multi-GNSS Uncombined Model Construction

After selecting the receiver clock of the GPS system as the reference, the G + C + R + E four-system fusion observation model can be obtained as follows:

(6) $$\left\{ \begin{array}{l} \Delta P_{r,i}^{j,G}=-\gamma_{r,i}^{j,G}\cdot \Delta r+c\cdot \delta {\tilde{t}}_{r}^{G}+M_{W}^{G}\cdot T_{w}+u_{i}^{G}\cdot {\tilde{I}}_{r,1}^{j,G}+\varepsilon_{i,P}^{j,G}\\ \Delta P_{r,i}^{j,C}=-\gamma_{r,i}^{j,C}\cdot \Delta r+c\cdot \delta {\tilde{t}}_{r}^{G}+c\cdot ISB_{r}^{C-G}+M_{W}^{C}\cdot T_{w}+u_{i}^{C}\cdot {\tilde{I}}_{r,1}^{j,C}+\varepsilon_{i,P}^{j,C}\\ \Delta P_{r,i}^{j,E}=-\gamma_{r,i}^{j,E}\cdot \Delta r+c\cdot \delta {\tilde{t}}_{r}^{G}+c\cdot ISB_{r}^{E-G}+M_{W}^{E}\cdot T_{w}+u_{i}^{E}\cdot {\tilde{I}}_{r,1}^{j,E}+\varepsilon_{i,P}^{j,E}\\ \Delta P_{r,1}^{j,R}=-\gamma_{r,1}^{j,R}\cdot \Delta r+c\cdot \delta {\tilde{t}}_{r}^{G}+c\cdot ISB_{r}^{R-G}+M_{W}^{R}\cdot T_{w}+u_{1}^{R}\cdot {\tilde{I}}_{r,1}^{j,R}+\varepsilon_{1,P}^{j,R}\\ \Delta P_{r,2}^{j,R}=-\gamma_{r,2}^{j,R}\cdot \Delta r+c\cdot \delta {\tilde{t}}_{r}^{G}+c\cdot ISB_{r}^{R-G}+M_{W}^{R}\cdot T_{w}+u_{2}^{s}\cdot {\tilde{I}}_{r,1}^{j,R}+c\cdot \eta^{j}\cdot \Pi_{r}^{R}+\varepsilon_{2,P}^{j,R} \end{array}\right.$$

(7) $$\left\{ \begin{array}{l} \Delta L_{r,i}^{j,G}=-\gamma_{r,i}^{j,G}\cdot \Delta r+c\cdot \delta {\tilde{t}}_{r}^{G}+M_{W}^{G}\cdot T_{w}+u_{i}^{G}\cdot {\tilde{I}}_{r,1}^{j,G}+\lambda_{i}^{G}\cdot {\tilde{M}}_{r,i}^{j,G}+\varepsilon_{i,L}^{j,G}\\ \Delta L_{r,i}^{j,C}=-\gamma_{r,i}^{j,C}\cdot \Delta r+c\cdot \delta {\tilde{t}}_{r}^{G}+c\cdot ISB_{r}^{C-G}+M_{W}^{C}\cdot T_{w}-u_{i}^{C}\cdot {\tilde{I}}_{r,1}^{j,C}+\lambda_{i}^{C}\cdot {\tilde{M}}_{r,i}^{j,C}+\varepsilon_{i,L}^{j,C}\\ \Delta L_{r,i}^{j,E}=-\gamma_{r,i}^{j,E}\cdot \Delta r+c\cdot \delta {\tilde{t}}_{r}^{G}+c\cdot ISB_{r}^{E-G}+M_{W}^{E}\cdot T_{w}-u_{i}^{E}\cdot {\tilde{I}}_{r,1}^{j,E}+\lambda_{i}^{E}\cdot {\tilde{M}}_{r,i}^{j,E}+\varepsilon_{i,L}^{j,E}\\ \Delta L_{r,i}^{j,R}=-\gamma_{r,i}^{j,R}\cdot \Delta r+c\cdot \delta {\tilde{t}}_{r}^{G}+c\cdot ISB_{r}^{R-G}+M_{W}^{R}\cdot T_{w}-u_{i}^{R}\cdot {\tilde{I}}_{r,1}^{j,R}+\lambda_{i}^{R}\cdot {\tilde{M}}_{r,i}^{j,R}+\varepsilon_{i,L}^{j,R} \end{array}\right.$$

From Equations (6) and (7), the specific forms of three groups ISB parameters can be determined as follows:

(8) $$\left\{ \begin{array}{l} ISB_{r}^{C-G}=dD^{C}-dD^{G}+D_{r,IF}^{C}-D_{r,IF}^{G}\\ ISB_{r}^{E-G}=dD^{E}-dD^{G}+D_{r,IF}^{E}-D_{r,IF}^{G}\\ ISB_{r}^{R-G}=dD^{R}-dD^{G}+D_{r,IF}^{R}-D_{r,IF}^{G} \end{array}\right.$$

From Equation (8), it is evident that ISB is primarily composed of two parts, i.e., the difference of hardware delay between different systems and the error term, which is independent of the receiver and is caused by the selection of the reference clock of different systems. The estimated parameters of the Equations (6) and (7) can be expressed as follows:

(9) $$X={\left[\begin{array}{cccccccccccccccccc} \delta x & \delta y & \delta z & \delta {\tilde{t}}_{r}^{G} & \Pi_{r}^{R} & T_{w} & ISB_{r}^{C-G} & ISB_{r}^{E-G} & ISB_{r}^{R-G} & {\tilde{I}}_{r,1}^{1,S} & \cdots & {\tilde{I}}_{r,1}^{m,S} & {\tilde{M}}_{r,1}^{1,S} & \cdots & {\tilde{M}}_{r,1}^{m,S} & {\tilde{M}}_{r,2}^{1,S} & \cdots & {\tilde{M}}_{r,2}^{m,S} \end{array}\right]}^{T}$$

### 2.3. ISB Parameter Processing Model

Typically, ISB parameters can be modeled using white noise, random constant and random walk processes. The white noise model is expressed as follows [22]:

(10) $$ISB_{r}^{S}(k)∼N\left(0,\sigma^{2}\right)$$

where k represents the epoch number. The advantages of the white noise model are (a) ISB parameters are irrelevant between epochs. (b) It is advantageous to adopt the white noise model when we are completely unaware of how the actual ISB parameters change or what constraint model to add [19].

The random constant model is relatively simple, i.e., an ISB parameter can be considered as a random constant and only one value is estimated in a day, which is expressed as follows:

(11) $$ISB_{r}^{S}(k)=ISB_{r}^{S}(k-1)$$

The random walk model [23] can be represented as:

(12) $$ISB_{r}^{S}(k)=ISB_{r}^{S}(k-1)+\varepsilon_{ISB},\begin{array}{cc} & \varepsilon_{ISB}∼N\left(0,\sigma_{\varepsilon_{ISB}}^{2}\right) \end{array}$$

The random walk model retains the estimate of the last epoch, and the variance increases linearly over time. After an experimental comparison of ISB parameters of G + C + R + E + J systems, Zhou et al., suggested that the white noise model or random walk model can be adopted in the PPP uncombined model process for a multi-system [19]. In this study, the white noise model is used to estimate ISB parameters.

## 3. Experimental Data and Processing Strategies

**Figure 1:**
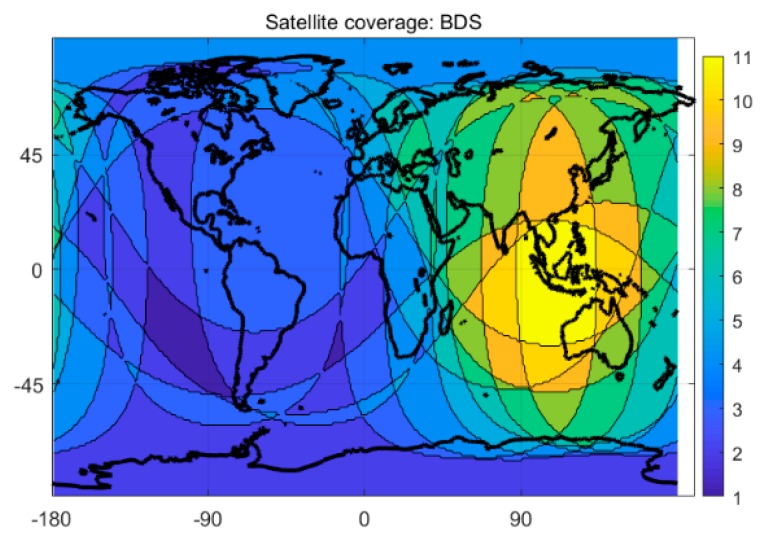
Number of Beidou system coverage satellites worldwide on day 150 in 2018.

**Figure 2:**
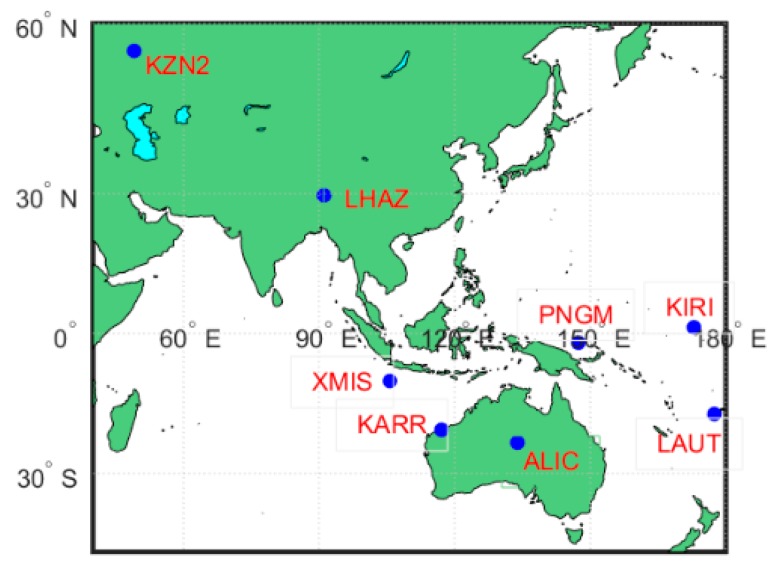
Station distribution.

To verify the model and analyse the ISB parameter characteristics, the data of eight MGEX stations for 28 days were selected (150th to 177th day in 2018). The criteria of the selection of the station is based on the following: (1) the station should be able to observe four-system satellites simultaneously. That is, the number of satellites observed by each system must be greater than 1 at the same time; (2) the reference coordinates of the station can be found in the file (gbm.clk). The Beidou second-generation satellites show poor visibility in the worldwide at present (Figure 1); thus, to satisfy the first requirement, most stations selected in this experiment were distributed in the Asia-Pacific region (Figure 2) to ensure that the tracking station could observe a sufficient number of BDS satellites. The observation data required pre-processing prior to the experiment. The precise reference coordinates of each station in the file (gbm.clk) were extracted to replace the prior coordinates in the observation file, and the coordinate bias in three directions (i.e., east, north and up) were obtained by filtering, which can be considered as positioning accuracy [28].

The receiver types in this experiment were primarily divided into two types, i.e., Leica and Trimble, while the antenna types were divided into three groups, i.e., leica25.r3, trm59,800.00 and javringant_dm, as shown in Table 1. A Kalman filter was used to estimate parameters day by day and station by station in the PPP data processing. The specific error correction and parameter estimation schemes are shown in Table 2.

## 4. Positioning Performance

### 4.1. DOP Value

**Figure 3:**
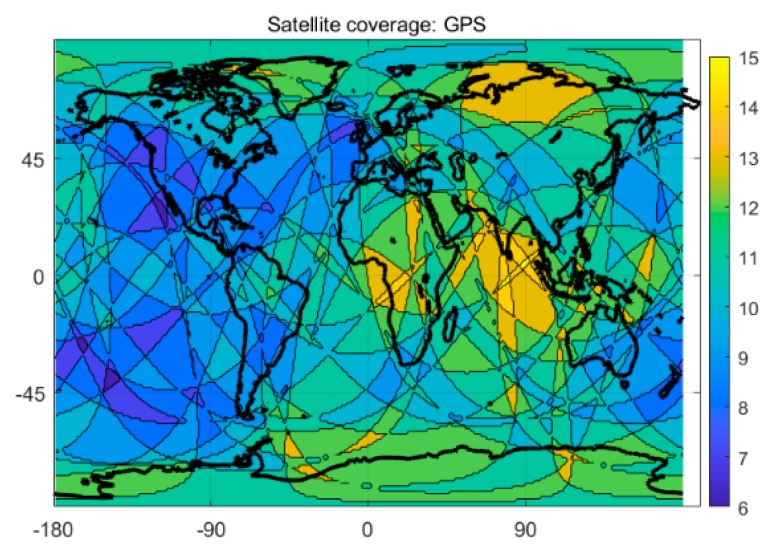
Satellite coverage of GPS system.

**Figure 4:**
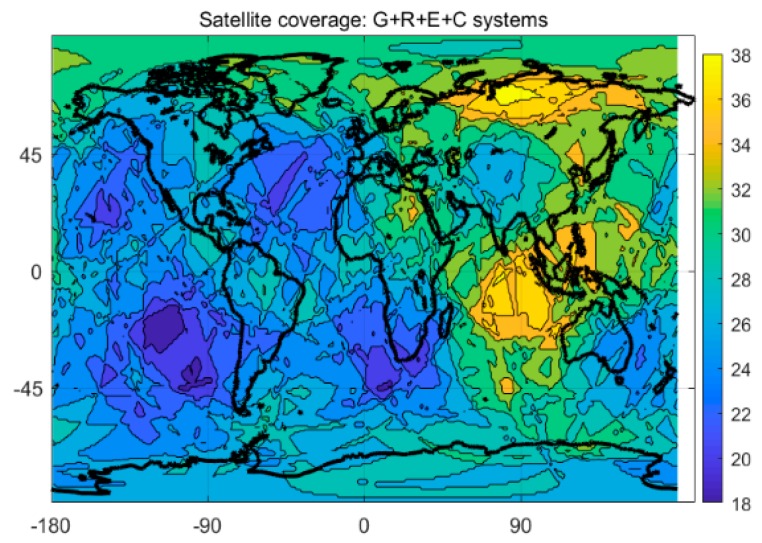
Satellite coverage of G + R + E + C system.

Compared to the single-system model, the multi-system model can provide more available satellites and better spatial geometry of the satellite constellation, which can theoretically yield significant improvement in positioning performance. Figure 3 and Figure 4 show the satellite coverage of the single GPS system and the G + C + R + E systems on a global scale at 0:0:00 on the 150th day of 2018. As shown in Figure 3, numbers of the satellite for a single GPS system range from 6 to 15, while the minimum number of available satellites for the G + C + R + E systems are 18, far more than the number of usable satellites provided by a single GPS system.

**Figure 5:**
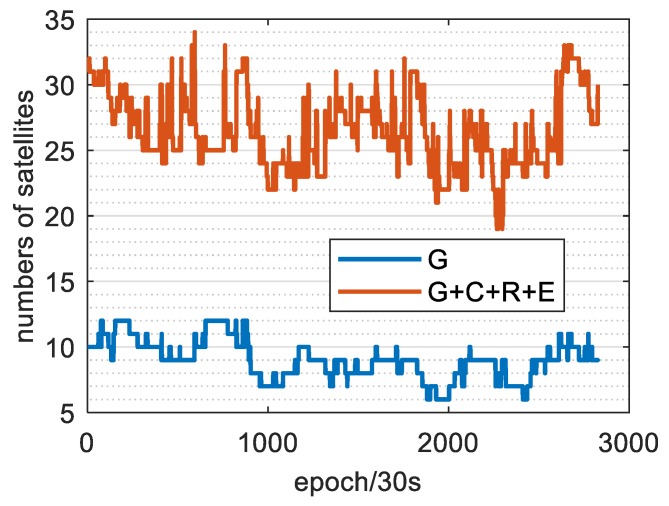
Available number of satellites for single GPS and G + C + R + E systems.

**Figure 6:**
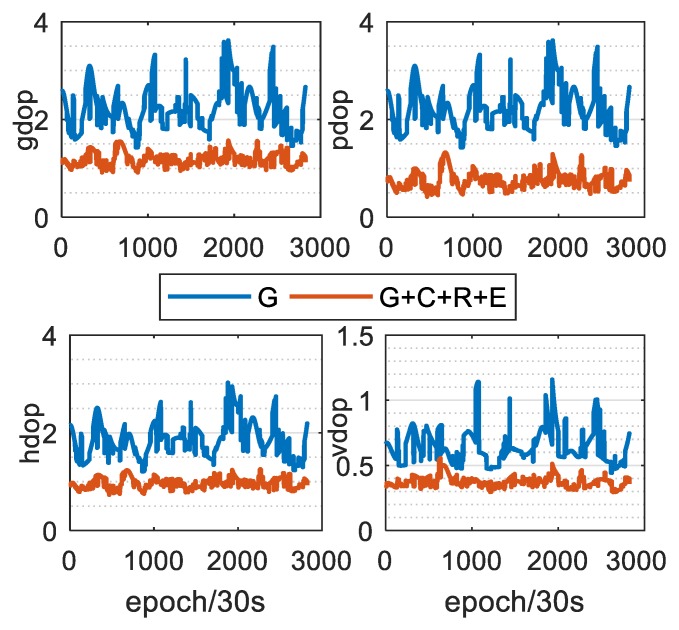
DOP values of single GPS and G + C + R + E systems.

Taking the XMIS station as an example, Figure 5 shows the number of available satellites of the single GPS and G + C + R + E systems on the same day. As can be seen, the number of satellites of the four systems on the same day reached 17–32, and the average number of available satellites was 26.1. However, the number of satellites in the single GPS system was 6–12, and the average number of usable satellites was 9, which is approximately one-third the number of usable satellites in the four systems. Figure 6 shows that the GDOP, PDOP, HDOP and VDOP values of the four systems, which are much less than that of the single GPS system. The average value decreased from 2.22, 2.97, 1.85 and 0.64 to 1.2, 0.77, 0.97 and 0.38, respectively. The increased number of available satellites effectively enhances the reliability and accuracy of PPP positioning.

### 4.2. Positioning Bias

**Figure 7:**
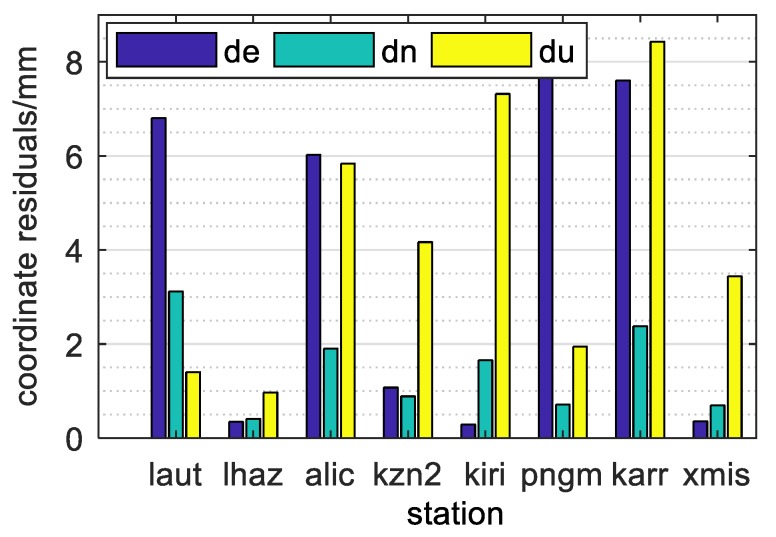
PPP results of day 150.

**Figure 8:**
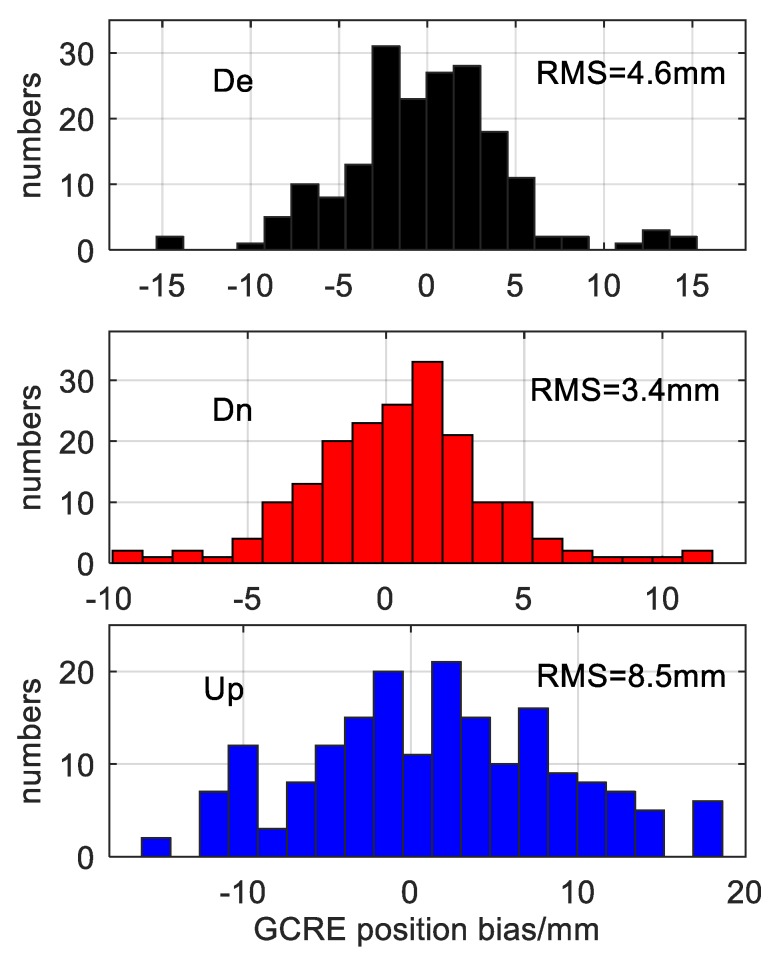
G + C + R + E system positioning error statistics.

Figure 7 shows that the coordinate bias of each station of the four-system model on the 150th day of 2018. The positioning biases in the E, N and U directions of all stations were at millimeter level, among which the positioning biases in the three directions of the lhaz station were all less than 1 mm. To further analyze the positioning of the uncombined PPP model, Figure 8 shows the coordinate bias statistics of all stations for 28 days, where Figure 8 denote the E, N and U directions, respectively, wherein the y axis indicates positioning bias in millimeters, and the ordinate indicates the number of stations. As can be seen, the RMS (Root Mean Square) of the positioning bias in the E, N and U directions were 4.6 mm, 3.4 mm and 8.5 mm, respectively. 

Therefore, we can conclude that more available satellites and better satellite constellation distribution (low DOP) can effectively improve the accuracy of PPP location solution. Moreover, a smaller RMS means a higher positioning accuracy and a more centralized positioning deviation distribution. Due to the correlation in PPP parameter estimation, the ultra-high precision position solution can better ensure the high accuracy and reliability of ISB parameter solution, which can better support the reliability and accuracy of the conclusion in the paper.

## 5. Estimation and Analysis of Inter-system Biases

According to the experimental arrangement of the manuscript, three groups of experiments are designed to study some clear characteristics of ISBs, which need to be verified by the measured data. The results of the experiments over 28 days showed very clear agreements.

### 5.1. Intra-day Variation of ISB

**Figure 9:**
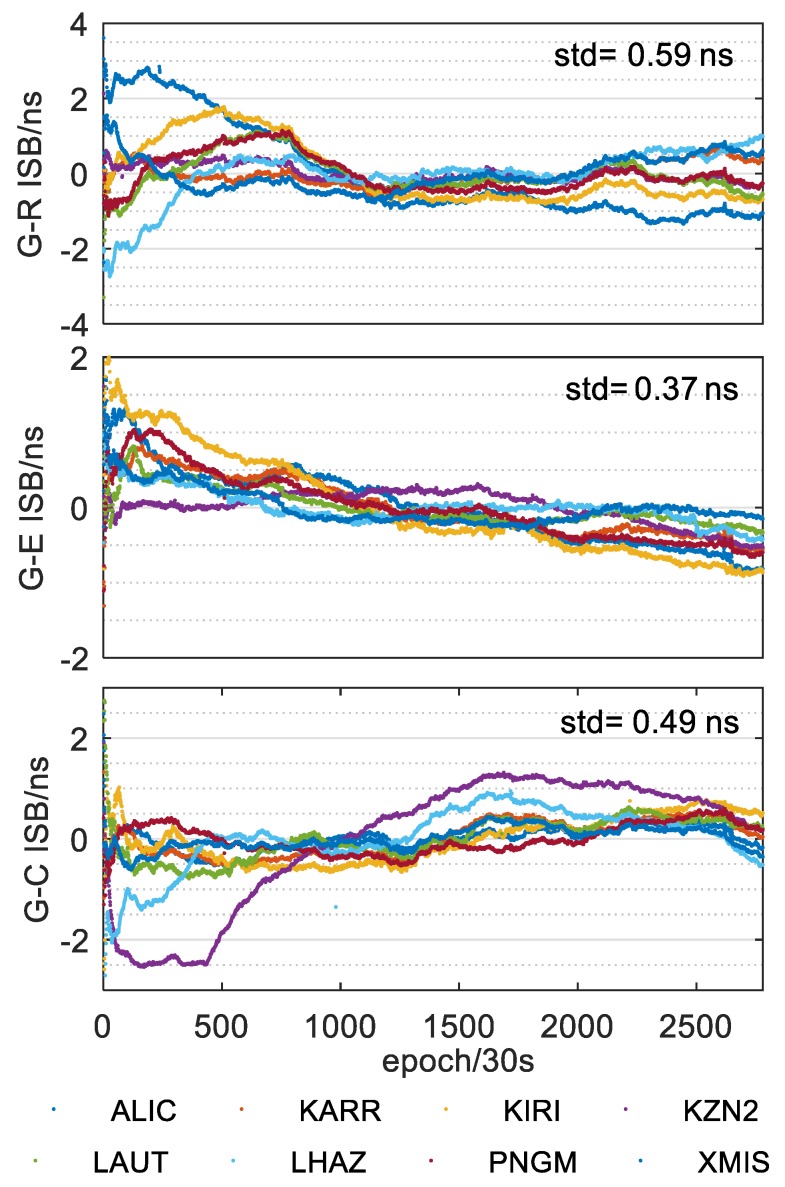
Fluctuation of inter-system bias (ISB) parameters of G-R, G-E and G-C on a single day (based on the respective mean values).

Through the epoch by epoch filtering calculation, the time series of inter-system bias were obtained, among them, G-R represents the inter-system bias between GPS and GLONASS, and G-E represents the inter-system bias between GPS and GALILEO, and G-C represents the inter-system bias between GPS and BDS.The intra-day variation of the ISB parameters was analyzed after determining their respective mean values. Figure 9 show the time series of the ISB parameters for the G-R, G-E and G-C systems of each station on the 150th day of 2018 (after deducting their mean values), respectively. As can be seen, the variation of the parameters among the three groups of ISB parameters was relatively stable within one day. The ISB parameter values of most stations were within ±1 ns, and the standard deviations of the three groups ISB were all less than 0.6 ns. Among them, the ISB parameter between the GALILEO and GPS systems were the most stable and the standard deviation was the smallest, which may be related to the GALILEO system’s good signal quality.

### 5.2. Inter-day Variation of ISB

**Figure 10:**
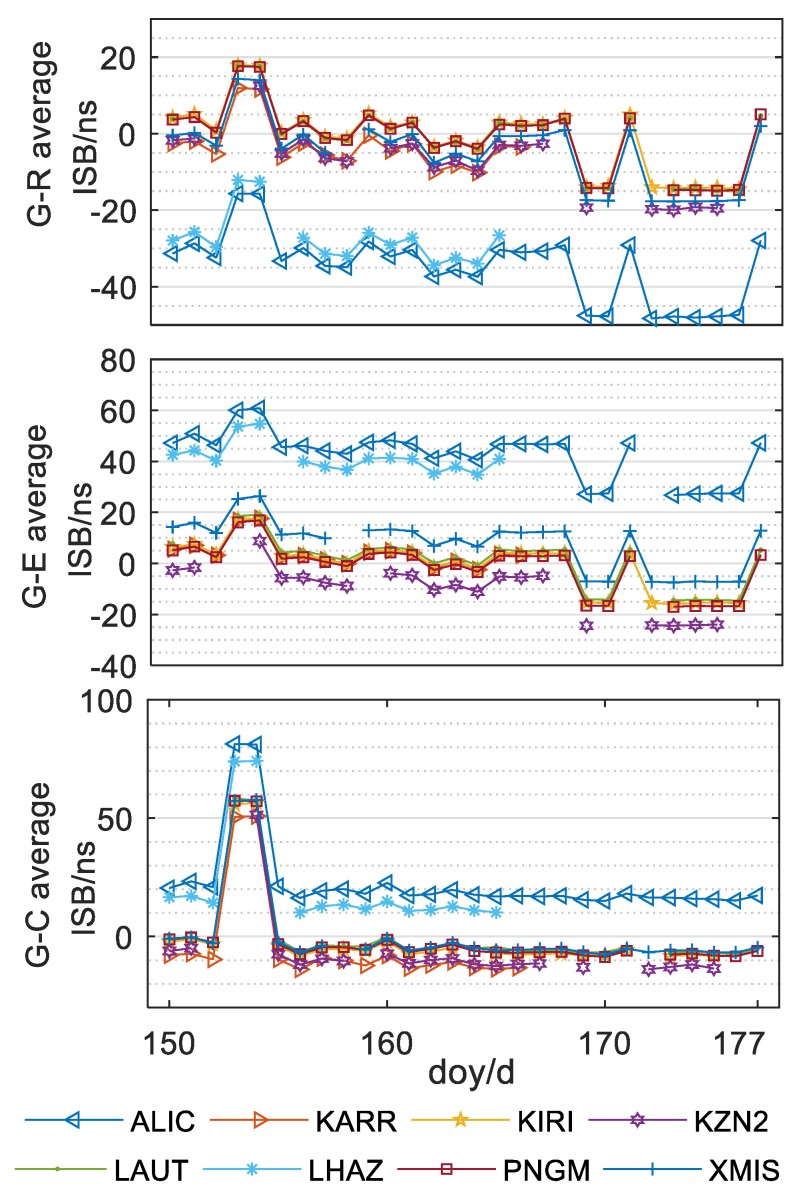
Time series of mean variation of ISB parameters of G-R, G-E and G-C.

**Figure 11:**
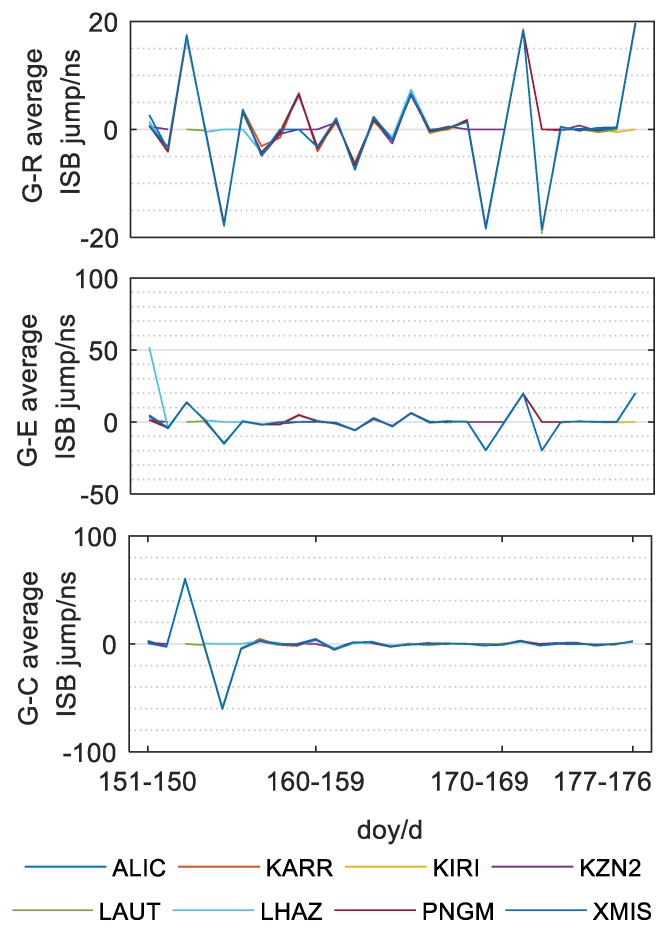
Jump of the mean ISB parameters of G-R, G-E and G-C in adjacent days.

After the analysis of the intra-day variation of ISB parameters, we concluded that the ISB parameters show high intra-day stability. To further analyze inter-day variation, the average values of the ISB parameters of all stations for 28 days (150–177th days of 2018) are shown in Figure 10 and the inter-day time series of the three sets of ISB parameters was obtained, as shown in Figure 11. Figure 10 and Figure 11 clearly show the distribution sequence of three groups of mean ISB parameters of these 8 stations in 28 days, which clearly reflects the following rules: 

(a) It can be seen from the figures that the ISB between BDS and GPS jumps on the 152th–153th day of the year of 2018, and the average value of the ISB in the following day is consistent with that in the 153th day. Then on the 154th–155th day of the year, it recovers around the average value of the ISB of before the 153th day. For more than 20 days after the 155th day, the ISB sequence fluctuated slightly.

(b) The ISB between GLONASS and GPS and the ISB between Galileo and GPS had obvious jump on the152th–153th, 153th–154th, 168th–169th, 170th–171th, 171th–172nd and 176th–177th days. Among them, the number of consecutive days of jump is at least 2 days, and the ISB value will not fluctuate significantly after the jump until the next jump.

(c) The jumping period of the ISB sequence between BDS and GPS is not identical with that of the other two groups of ISB sequences. Except for the two-day jumping, there is no obvious fluctuation in other days. This may be related to the BDS receiver, and the specific reasons need to be further studied.

(d) In general, the average value of the ISB of single-day solution of each station is not stable in some time period, and the amplitude of jump is irregular (up to 60 ns).

(e) The ISB parameters of all stations show similar variation trends and amplitude on the same day. This situation is independent of receiver, which may be ascribed to a satellite reference clock of a different system (Wang et al. 2018).

### 5.3. Correlation between ISB and Receiver Type

**Figure 12:**
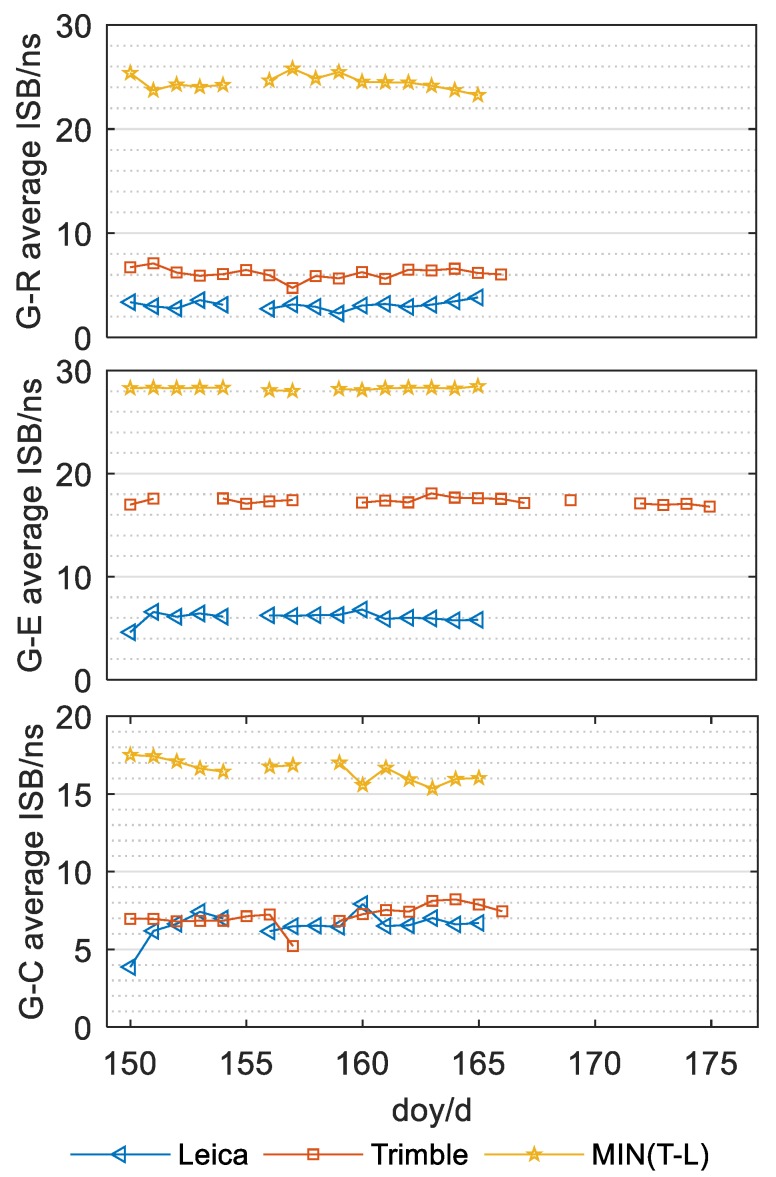
Relationship between ISB parameters and receiver types.

According to the analysis of Equation (8), the ISB parameters were related to both the receiver clock and receiver hardware delay. Thus, it is necessary to further analyze the correlation between the parameters of inter-system bias and receiver type. According to the types of receivers shown in Table 1, the receivers of the eight stations in this experiment were divided into two types, i.e., the allic and lhaz stations have the Leica gr25 receiver type, and the karr, kiri, kzn2, laut, pngm and xmis stations have the Trimble netr9 receiver. The relationship between the ISB parameters and different receiver types is shown in Figure 12.

In Figure 12, the y axis represents the difference values of the three groups of ISB parameters of two different stations, respectively, and the x axis indicates the target day. Corresponding to three different types of ISB parameters, it should select two stations with the largest or smallest mean value of the single-day solution of the ISB parameter for each receiver type, and, in the process, due to the missing observation values on certain days from some stations, for fairness, it is considered that the ISB parameters of the corresponding type of measuring station are still the largest or smallest in the subsequent missing days. Blue triangles in Figure 12 represent the difference between the maximum and minimum ISB mean values of stations equipped with Leica type receivers in each day, and red Squares represent the difference between the maximum and minimum ISB mean the values of stations equipped with Trimble type receivers in each day. The yellow pentagonal star in Figure 12 shows the minimum difference between ISB mean values of stations equipped with Trimble type receivers and ISB mean values of stations equipped with LECIA type receivers in each day. As shown in Figure 12, apart from the graph for the ISB_GE parameter, the maximum change in amplitude of the mean single-day ISB value between two stations with the same type of receiver were all less than 9 ns, but the minimum change between two mean values of the single-day solutions corresponding to two stations equipped with two different kinds of receivers were all greater than 15 ns. Even considering the ISB_GE parameter, the minimum difference value of the mean values of the single-day solutions of the ISB parameters between two stations equipped with different kinds of receivers was far greater than those equipped with the same receiver. Based on the analysis shown in Figure 12, the mean value of the single-day solutions of ISB parameters between two stations equipped with the same kind of receiver demonstrates a small change range, and the change is relatively stable; however, it varies significantly for two stations equipped with different types of receivers. Therefore, we conclude that there is a clear relationship between the ISB parameters and receiver type.

## 6. Conclusions

This paper focused on the estimation and analysis of the ISB parameters based on the uncombined PPP using the GLONASS, BDS, GALILEO and GPS systems. We first deduced the uncombined PPP function model of G + C + R + E four-system fusion processing. Aiming at the problem that there are much many parameters to be evaluated for the four system uncombined PPP and the inter-frequency deviation of the pseudo range of GLONASS system, we separate the pseudo range IFB parameter of GLONASS system of single epoch through theoretical derivation, simplify the PPP function model, improve the model strength and parameter estimation efficiency. And we then determined the processing model of the ISB parameters. Next, we used the MGEX station’s 28-day data to estimate and analyze three kinds of ISB parameters. Our conclusions are summarized as follows.

1. The uncombined PPP of the G + C + R + E system fusion provides high positioning accuracy. From the statistical results of the 28-day data, the rms of positioning deviation in the E, N and U directions reached 4.6 mm, 3.4 mm and 8.5 mm, respectively, which can effectively guarantee the reliability and accuracy of the ISB parameter solution.
2. The intra-day variation of ISB was relatively stable, and the standard deviation of three kinds of ISB parameters were all less than 0.6 ns. Among them, the ISB parameter between the GALILEO system and GPS system was the most stable, and the standard deviation was the smallest, which may be related to the GALILEO system’s good signal quality.
3. The mean ISB parameters value of single-day solution of each station is not stable in the time period, and the amplitude of jump is irregular (up to 60 ns); however, the ISB parameters of all stations show similar variation trends and amplitude on the same day. This situation is independent of receiver, which may be ascribed to satellite reference clock of different system.
4. There is a clear relationship between the ISB parameter and receiver type.

We acknowledge the following limitations in this study. The receiver types of all MGEX stations have not been analyzed statistically, and the accurate relationship between the ISB parameters and receiver type have not been analyzed systematically. These issues will be the focus of future work.

## Figures and Tables

**Table 1:** Receiver and receiver antenna types of stations.

Station	Types of Receiver	Types of Receiver Antenna
alic	leica gr25	leica25.R3
karr	trimble netr9	trm59,800.00
kiri	trimble netr9	trm59,800.00
kzn2	trimble netr9	trm59,800.00
laut	trimble netr9	javringant_dm
lhaz	leica gr25	leica25.R4
pngm	trimble netr9	trm59,800.00
xmis	trimble netr9	javringant_dm

**Table 2:** Precise point positioning (PPP) parameter estimation and related error term processing strategies.

Project	Processing Strategy
Observed value	BDS, GLO, GPA, GAL pseudorange and carrier phase raw observations
Sampling interval	30s
Signal	BDS:B1,B2 GLO:L1,L2 GPS:L1,L2 GAL:E1,E5a
Parameter estimation	Kalman filtering
Height cut-off Angle	8°
Weight distribution of observed values	Height angle model
Tides	(ocean tide, earth tide, polar tide) model correction
Satellite antenna phase center correction	BDS: Adopted the recommended values of ESA as pco and pcv values; GALILEO: Adopted the recommended values of ESA as pco and pcv values; GPS: Use the recommended values in the igs14.ATX file as pco and pcv values; GLONASS: Use the recommended values in the igs14.ATX file as pco and pcv values
Receiver antenna phase center correction	BDS: Use GPS recommended values in igs14.ATX file as pco and pcv values; GALILEO: Use GPS recommended values in igs14.ATX file as pco and pcv values; GPS: Use the recommended values in the igs14.ATX file as pco and pcv values; GLONASS: Use the recommended values in the igs14.ATX file as pco and pcv values
Satellite ephemeris and clocks files	Adopt the precision ephemeris and precision clock difference products provided by GBM
Tropospheric delay	Model correction + random walk
Ionospheric delay	White noise estimation
Receiver coordinates	Estimate as a constant in one day
Ambiguity	Estimate as a constant in one day
Receiver clock	Random walk
ISB	White noise estimation
